# Cloning and expression of a *Trichinella spiralis* putative glutathione S-transferase and its elicited protective immunity against challenge infections

**DOI:** 10.1186/s13071-017-2384-1

**Published:** 2017-09-29

**Authors:** Chun Ying Liu, Yan Yan Song, Hua Na Ren, Ge Ge Sun, Ruo Dan Liu, Peng Jiang, Shao Rong Long, Xi Zhang, Zhong Quan Wang, Jing Cui

**Affiliations:** Department of Parasitology, School of Basic Medical Sciences, Zhengzhou University, Zhengzhou, 450052 China

**Keywords:** *Trichinella spiralis*, Glutathione-S-transferase (GST), Protective immunity, Mice

## Abstract

**Background:**

Glutathione-S-transferase (GST) is a widespread multigene family of detoxification enzymes. The vaccination of mice with recombinant GST of 24 kDa from *Trichinella spiralis* elicited a low immune protection against challenge infection. The objective of this study was to characterize the *T. spiralis* putative GST gene (*TspGST*) encoding a 30.8 kDa protein and to evaluate its potential as a candidate antigen for anti-*Trichinella* vaccine.

**Methods:**

The full-length cDNA sequence of *TspGST* from *T. spiralis* muscle larvae (ML) was expressed in *E. coli*. The enzymatic activity and antigenicity of the rTspGST were identified by spectrophotometry, Western blot, and ELISA. The expression of *TspGST* at *T. spiralis* various stages was investigated by RT-PCR and indirect immunofluorescent test (IIFT). Serum level of total IgG, IgG1, and IgG2a antibodies against rTspGST were measured by ELISA. The immune protection produced by vaccination with rTspGST against *T. spiralis* was evaluated.

**Results:**

The sequencing results showed that the cDNA of *TspGST* was 840 bp, and encoded a protein of 279 amino acids, which had a molecular size of 30.8 kDa and a pI of 5.21. Its amino acid sequence shares 37% similarity with TsGST. The rTspGST protein had enzymatic activity of GST. On Western blot and ELISA analysis, the native TspGST protein with 30.8 kDa in crude antigens derived from adult worms (AW), newborn larvae (NBL), infective intestinal larvae (IIL) and ML was recognized by anti-rTspGST sera, but the ML ES antigens could be not recognized by anti-rTspGST sera. Expression of *TspGST* was found in all of *T. spiralis* various stages (AW, NBL, ML, and IIL). An immunolocalization analysis identified TspGST in different stages (mainly in cuticles) of the nematode. The mice vaccinated with the rTspGST elicited Th2-predominant immune responses, showed a 34.38% reduction of adult worms and a 43.70% reduction of muscle larvae.

**Conclusions:**

Immunization with rTspGST produced a partial immune protection, and the rTspGST could be regarded as a potential candidate target for an anti-*Trichinella* vaccine.

## Background

Trichinellosis is a major foodborne parasitic disease resulted from ingesting raw or semi-cooked meat infected with *Trichinella* infective larvae [1]. *Trichinella* infection in animals and humans has been recorded in most provinces on the Chinese Mainland, and 15 outbreaks of trichinellosis consisting of 1387 cases and four deaths occurred from 2004 to 2009 [2–4]. Pork is the dominating source of infection for trichinellosis outbreaks in China. Swine *Trichinella* infection is an important hygiene problem for meat product safety and public health [5]. Therefore, an anti-*Trichinella* vaccine for domestic pigs has become another promising measure to prevent the transmission of trichinellosis from swine to humans [6–9].

After being ingested, *Trichinella spiralis* muscle larvae (ML) are released in the stomach with the help of digestive enzymes, migrate to the intestine, and develop into the infective intestinal larvae (IIL) [10]. The IIL penetrate the intestinal epithelium, molt four times, and then develop into adult worms (AW) which copulate and produce newborn larvae (NBL). Since the IIL is the first invasion phase during *T. spiralis* life-cycle, an anti-*Trichinella* vaccine against the IIL could potentially prevent the intestinal *Trichinella* infection, which might block or inhibit the subsequent occurrence of the muscular phase of trichinellosis. Previous studies demonstrated that the *T. spiralis* glutathione-S-transferase (TsGST) gene (GenBank: XM_003371707.1) encoding a 24 kDa protein was an up-regulated gene in the IIL compared with the ML stage [11, 12], suggesting that the TsGST might be a larval invasion-related protease. However, the vaccination of mice with the rTsGST resulted in a 35.71% reduction of adults in the intestine and a 38.55% reduction of larvae in muscle, indicating that immunization with the rTsGST elicited a low immune protection against *T. spiralis* infection [13]. In this study, we cloned and expressed a putative *T. spiralis* GST gene (GenBank: XM_003373603) encoding a 30.8 kDa protein [14], the characteristics of TspGST was investigated, and the immune protection generated by the rTspGST immunization was evaluated in mice.

## Methods

### Parasites and experimental animals

The *T. spiralis* isolate (ISS534) used here was from a domestic pig in the Henan Province of China. It was passaged in Kunming mice at 6–8 month intervals in our laboratory. Female BALB/c mice, specific pathogen-free (SPF) and 4–6 weeks of age, were obtained from the Henan Provincial Experimental Animal Center (Zhengzhou, China).

### Parasite collection and antigen preparation

The mice were infected orally with 300 *T. spiralis* ML and euthanized at 42 days post infection (dpi). The carcasses were artificially digested by digestion solution containing 0.33% pepsin (1:31,000; Sigma-Aldrich, St. Louis, MO, USA) and 1% HCl at 43 °C for 2 h, and then the ML were collected [15, 16]. The IIL were recovered from small intestines of experimentally infected mice at 6 h post infection (hpi) [17], and the AW were respectively collected from the duodenum and jejunum of infected mice at 3 and 6 dpi [18]. The NBL was obtained from female worms incubated in RPMI-1640 medium at 37 °C for 24 h [19]. The soluble proteins (crude antigens) of AW, NBL, ML and IIL, and excretory-secretory (ES) proteins from the ML were prepared [20, 21].

### Cloning, expression, and identification of TspGST

The *TspGST* gene (GenBank accession no. XM_003373603) was amplified by PCR using specific primers with *Bam*HI and *Hind* III restriction enzyme sites (underlined) (forward: 5′-TAT AGG ATC CAT GAC CAA CAC GTC GAA GAA AGG-3′; reverse, 5′-GCC CAA GCT TTC ATT GAC TTT CAA TAG TCA CTG G-3′). The purified PCR product was cloned into the pMD19-T vector (Takara, Dalian, China), subsequently sub-cloned into the pQE-80 L (Novagen, La Jolla, CA, USA). The recombinant plasmid carrying the *TspGST* gene was transformed into *Escherichia coli* BL21 (DE3) (Novagen), and expressed under IPTG induction. The rTspGST was purified using Ni-NTA-Sefinose resin (Sangon Biotech, Shanghai, China). The concentration of the purified rTspGST was assayed as described previously [22], and identified by SDS-PAGE analysis [23]. The gel was stained with 0.25% Coomassie brilliant blue R-250 (Sigma-Aldrich), and subsequently decolorized.

### Assay of rTspGST enzymatic activity

The enzymatic activity of the purified rTspGST was determined spectrophotometrically by observing the production of the thioether (S-2,4-dinitrophenylglutathione) between the reduced form of glutathione and 1-chloro-2,4-dinitrobenzene (CDNB; Sigma-Aldrich). The enzymatic product extinction coefficient ε = 9600 M^-1^ cm^-1^ at 25 °C at 340 nm. The reaction was conducted in 1 ml reaction mixture consisting of 1 mM reduced GSH, 7 μg of rTspGST, and 100 mM phosphate buffer. The reaction was started by the addition of 1 mM CDNB as substrates, and the increase in absorbance at 340 nm was recorded for 5 min [13]. The rTsGST samples were repeatedly assayed three times, and the results were shown as mean values ± standard deviation (SD).

### Immunization of mice

The mice were randomly divided into three groups (20 animals per group). The vaccination group of mice was subcutaneously inoculated at multiple abdominal sites with 20 μg of rTspGST emulsified with complete Freund’s adjuvant and boosted three times with the rTspGST with incomplete Freund’s adjuvant at an interval of 10 days. Two control groups were inoculated with adjuvant or PBS using the vaccination mentioned above procedure [24]. Tail blood from vaccinated mice was respectively collected at 0, 10, 20, 30 and 40 days after vaccination.

### Determination of antibodies to rTspGST

Serum anti-rTspGST IgG of vaccinated mice was assayed by ELISA with crude antigens of ML, IIL, AW and NBL, ML ES antigens, or the rTsGST; the IgG subtypes (IgG1 and IgG2a) were also assayed by ELISA with the rTspGST [25]. The plates (Nunc, Roskilde, Denmark) were coated at 4 °C overnight with 1.5 μg/ml of the rTspGST, and crude antigens of *T. spirali* various stages. The plates were blocked with 200 μl of PBS-Tween 20 (PBST) containing 5% skimmed milk. Serum samples diluted at 1:100 were added and incubated at 37 °C for 1 h. HRP-conjugated goat anti-mouse IgG, IgG1 or IgG2a (1:5000; Sigma-Aldrich) were used as the secondary antibodies, and o-phenylenediamine dihydrochloride (OPD; Sigma-Aldrich) was used as a substrate. The absorbance at 490 nm was assayed with a microplate reader (Tecan, Schweiz, AG, Switzerland).

### Western blot analysis

Proteins included 15 μg/lane of ML crude and ES antigens, and the rTsGST. The proteins were separated by SDS-PAGE at 120 V for 2.5 h, then transferred onto nitrocellulose membranes (Merck Millipore, Billerica, MA, USA) at 20 V for 40 min [26]. The membranes were clipped into strips, blocked by PBST with 5% skimmed milk at 37 °C for 1 h, and incubated at 37 °C for 1 h with 1:100 dilutions of different mouse sera (anti-rTspGST sera, sera from mice infected *T. spiralis* at 42 dpi and normal mouse sera). After washing, the strips were incubated with HRP-conjugated goat anti-mouse IgG (Sigma-Aldrich). 3, 3′-diaminobenzidine tetrahydrochloride (DAB; Sigma-Aldrich) was used as the substrate [13].

### RT-PCR analysis of TspGST gene transcription

Total RNA was respectively extracted by using Trizol reagent (Invitrogen, Carlsbad, CA, USA) from *T. spiralis* various stages (AW at 3 and 6 dpi, NBL, ML, and IIL). RT-PCR was conducted as described [27]. A housekeeping gene of *T. spiralis* (glyceraldehyde-3-phosphate dehydrogenase, GAPDH) was also amplified as a positive control. PBS was used as negative control for all PCRs.

### Indirect immunofluorescent test (IIFT)

The IIFT was used to determine the expression and immunolocalization of the native TspGST at *T. spiralis* diverse stages [28]. The whole intact worms and sections of the parasite tissues were blocked in PBS containing 5% goat sera, reacted with 1:100 dilutions of anti-rTspGST sera, infection sera or normal mouse sera at 37 °C for 1 h. The FITC-labeled goat anti-mouse IgG (Santa Cruz Biotechnology, Dallas, Texas, USA) diluted at 1:100 was used as the secondary antibodies. After washing, the whole worms and tissue sections were observed with a fluorescent microscope (Olympus, Tokyo, Japan).

### Challenge experiment

Ten days after the last vaccination, each mouse of three groups was challenged orally with 300 *T. spiralis* ML. Ten mice of each group were sacrificed at 5 dpi, and the intestinal AW were recovered and counted [29, 30]. The ML burdens from the other ten mice of each group were investigated at 42 dpi by artificial digestion [13, 31]. The immune protection was evaluated as the reduction rate of recovered AW and larvae per gram (LPG) of muscles of the vaccinated groups versus that of the PBS group [32].

### Statistical analysis

The statistical analyses of the data were performed via SPSS for Windows, version 17.0. The data of OD values of antibody levels and the AW and ML recovery were shown as the mean value ± standard deviation (SD). One-way ANOVA or Student’s t-test was utilized for the analysis of intra- and intergroup differences, and *P* < 0.05 was regarded as statistically significant.

## Results

### Molecular cloning and expression of the cDNA encoding a 30.8 kDa TspGST

**Fig. 1:**
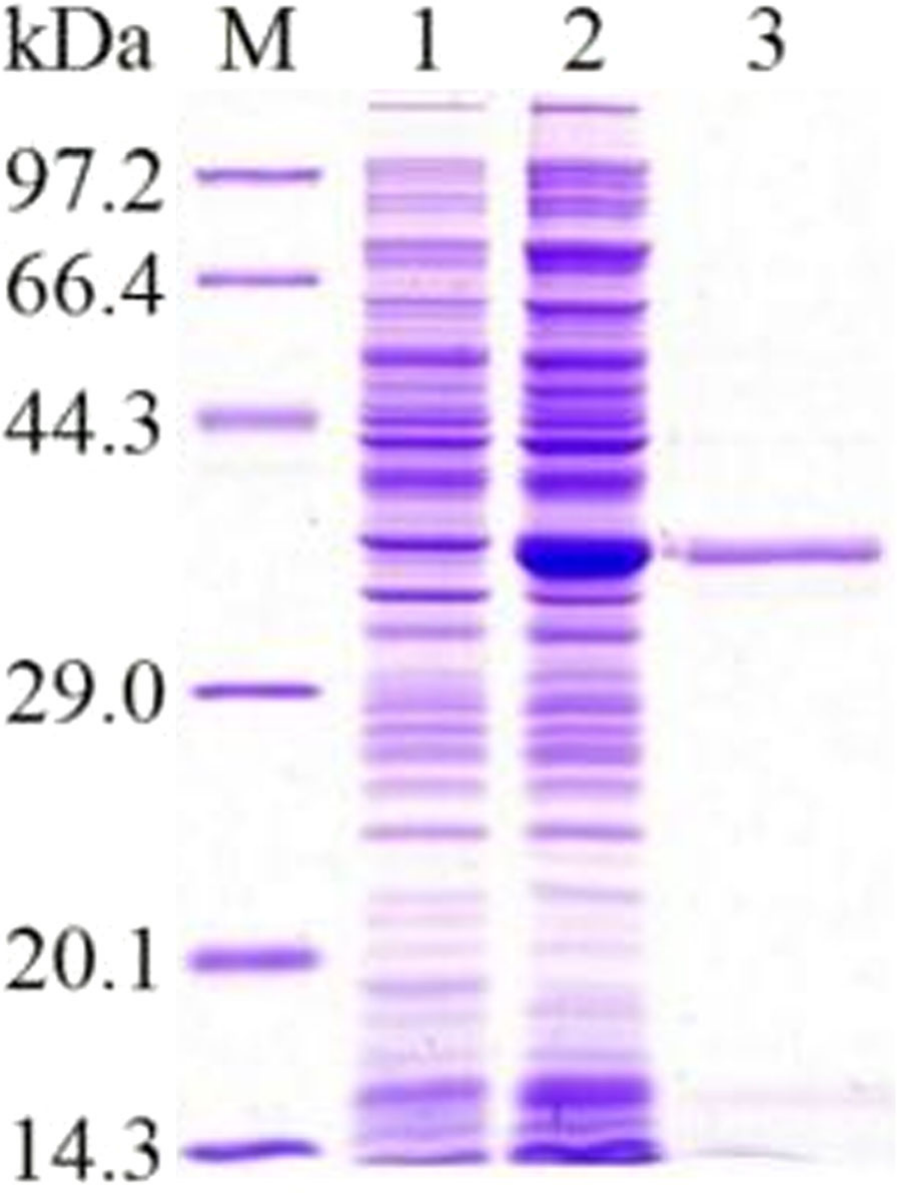
SDS-PAGE analysis of the rTspGST. Lane M: low molecular weight protein marker; Lane 1: the lysis of the recombinant bacteria; Lane 2: the lysis of the induced recombinant bacteria; Lane 3: rTspGST purified by Ni-NTA-Sefinose column

The full-length TspGST cDNA was 840 bp and encoded a protein of 279 amino acids, which had a molecular size of 30.8 kDa and a pI of 5.21. SignalP 4.1 Server predicted that the TspGST did not have signal peptides. The TspGST had only the 37% identity with TsGST at the amino acid level, which was aligned by protein BLAST. The pQE-80 L harboring the TspGST gene was successfully transformed into *E. coli* BL21 (DE3) bacteria. After being induced with 0.5 mM IPTG, recombinant TspGST produced a band of approximately 30.8 kDa protein examined by SDS-PAGE, the molecular weight of the rTspGST protein was compatible with its predicted size. On SDS-PAGE analysis, the rTspGST protein purified by Ni-NTA-Sefinose Column showed a clear single band (Fig. 1).

### Enzymatic activity of rTspGST

The enzymatic activity analysis revealed that the enzymatic activity of the rTspGST to conjugate glutathione to CDNB was 115.42 ± 7.71 U/mg protein.

### Analysis of rTspGST antigenicity

**Fig. 2:**
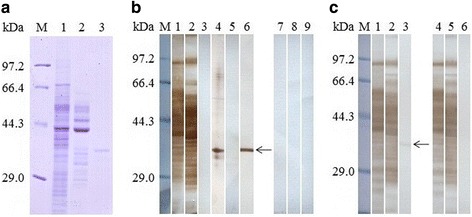
Identification of the rTspGST. **a** SDS-PAGE analysis of *T. spiralis* ML crude antigen (Lane 1), ES antigen (Lane 2) and rTspGST (Lane 3). **b** Western blot analysis of rTspGST antigenicity. *T. spiralis* ML crude antigens (Lane 1) and ES antigens (Lane 2) were probed by sera of mice infected with *T. spiralis* at 42 dpi, but the rTsGST (Lane 3) was not probed by infection sera. The native TspGST protein in ML crude protein (Lane 4) and rTspGST (Lane 6) were probed by anti-rTspGST sera, but the ML ES antigens (Lane 5) were not probed by anti-TspGST sera. *T. spiralis* ML crude antigens (Lane 7), ES antigens (Lane 8) and rTspGST (Lane 9) were not probed by normal mouse sera. **c** Western blot analysis indicated that *T. spiralis* ML crude antigens (Lane 1), ES antigens (Lane 2) and rTspGST (Lane 3) were recognized by sera of mice immunized with ML crude antigens. The ML crude protein (Lane 4) and ES antigens (Lane 5) were recognized by sera of mice immunized with ML ES antigens, but rTspGST (Lane 6) were not recognized by sera of mice immunized with ML ES antigens

**Fig. 3:**
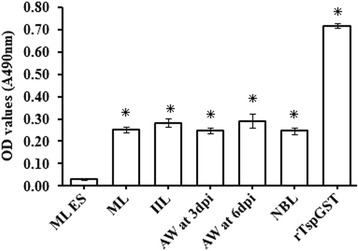
Serum-specific anti-rTspGST IgG level of mice vaccinated with rTspGST determined by ELISA with crude antigens of *Trichinella spiralis* various stages (AW at 3 and 6 dpi, NBL, ML and IIL) and ML ES antigens. The OD values shown for each group are the mean ± SD of the antibody levels from ten mice. Asterisks indicate statistically significant differences (*P < 0.01)

The rTspGST was identified by anti-rTspGST serum on Western blot analysis. Anti-rTspGST sera also identified the native TspGST protein in ML crude antigens but did not identify the ML ES antigens. Furthermore, the rTspGST was identified by sera of mice vaccinated with ML crude antigens, but not by sera of mice vaccinated with ML ES antigens (Fig. 2). The results were obtained under the current experimental conditions, and it is possible that the TspGST cannot be detected if the expression of *TspGST* in ML ES antigens is low. Also, the ELISA results showed anti-rTspGST sera was strongly reacted with the rTspGST, and crude antigens of *T. spiralis* different stages (NBL, AW at 3 and 6 dpi, ML and IIL), but anti-rTspGST sera did not react with the ML ES antigens (Fig. 3). These results suggested that TspGST would be one component of the somatic proteins of *T. spiralis* different phases, but not from ML ES proteins, which indicates that TspGST would not be secreted, and did not expose to the immune system during the muscular phase of *T. spiralis* infection.

### RT-PCR analysis of TspGST gene transcription at various stages

**Fig. 4:**
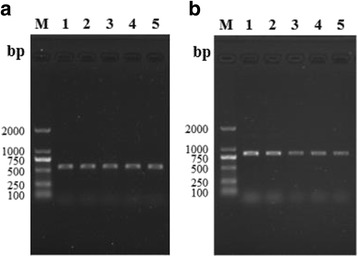
RT-PCR analysis of GAPDH gene **a** and TspGST gene **b** at *Trichinella spiralis* various development stages. Lane M: DL2000 DNA marker; Lane 1: ML; Lane 2: IIL; Lane 3: AW at 3 dpi; Lane 4: AW at 6 dpi; Lane 5: NBL

The *TspGST* gene (840 bp) was amplified by RT-PCR from *T. spiralis* various stages (AW at 3 and 6 dpi, NBL, ML and IIL). The housekeeping GAPDH gene was also amplified from all *T. spiralis* developmental stages as a positive control (570 bp) (Fig. 4).

### Expression and immunolocalization of TspGST by IIFT

**Fig. 5:**
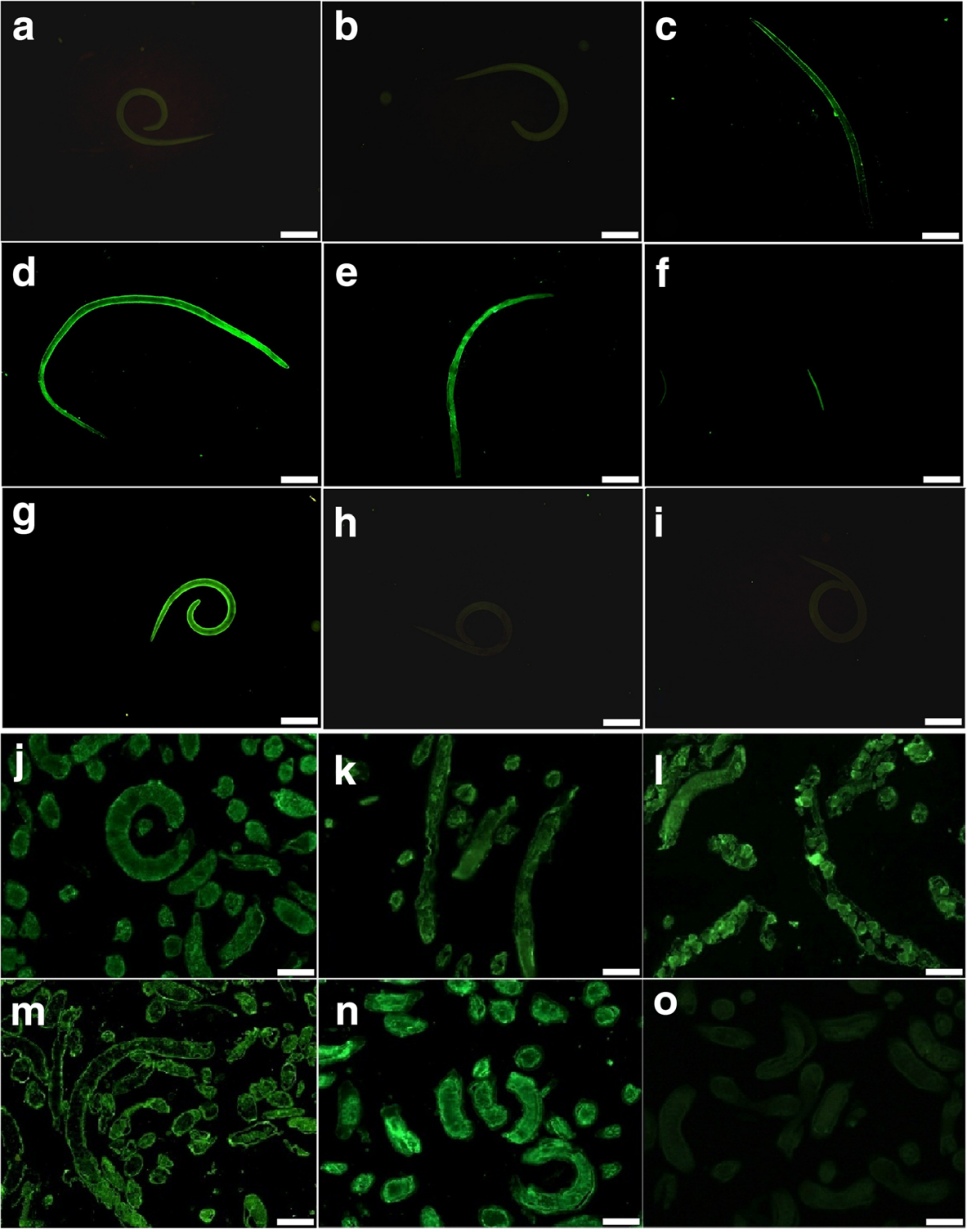
Expression and immunolocalization of TspGST at *Trichinella spiralis* various stages. **a**-**i** The results of IIFT with the intact parasites probed by anti-rTspGST sera. The obvious fluorescent staining is observed on the surface of AW at 3 (**c**) and 6 dpi (**d**, **e**), and NBL (**f)**, but not on the surface of ML (**a**) and IIL (**b)**. The ML recognized by sera from *T. spiralis* experimentally infected mice g was used as a positive control; the ML incubated with normal mouse sera (**h**), and PBS (**i**) were used as negative controls. **j**-**o** Sections of intact worms (ML, IIL and AW) reacted with anti-rTspGST sera. The immunostaining is observed at the cuticle of ML (**j**), IIL (**k**), AW at 3 (**l**) and 6 dpi (**m**), especially at embryos within the female uterus. The ML recognized by sera from *T. spiralis* experimentally infected mice n as positive control; the ML incubated with normal mouse sera o as negative control. *Scale-bars*: **a**-**e**, **g**-**i**, 200 μm; **f**, **j**-**o**, 100 μm

The IIFT with the intact parasite revealed that green fluorescence staining by anti-rTspGST sera was seen on the surface of AW at 3 and 6 dpi as well as NBL. In contrast, no visible staining was found on the surface of the ML and IIL (Fig. 5). After the worm tissue sections from *T. spiralis* different phases were probed with anti-rTspGST sera, the positive staining was distributed at the cuticles of the AW, ML and IIL, and the embryos within the female uterus.

### Humoral antibody responses elicited by vaccination with rTspGST

**Fig. 6:**
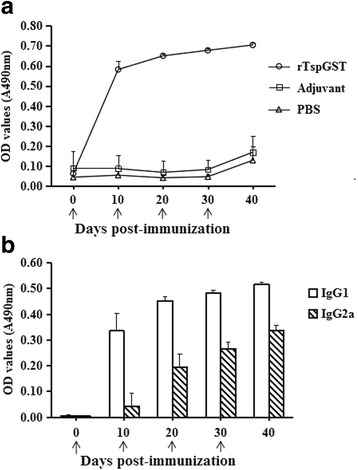
Antibody responses in mice immunization with rTspGST. **a** Specific anti-rTspGST IgG levels in the sera of vaccinated mice at different time intervals after vaccination. **b** Serum levels of the subclasses IgG1 and IgG2a. The OD values shown for each group are the mean ± SD of the antibody levels from 10 animals. The vaccination time points are marked with an arrow (↑)

Serum specific anti-rTspGST IgG, IgG1, and IgG2a at different time after vaccination were examined by ELISA with the rTspGST. The levels of anti-rTspGST IgG in vaccinated mice were obviously elevated after the first and second vaccination and continued to rise at the end of the experiment (Fig. 6). But all of the mice injected with PBS or adjuvant did not show evidently anti-rTspGST antibody responses. Anti-rTspGST IgG subtype assay demonstrated that after the first and second vaccination, the IgG1 levels were remarkably higher than the IgG2a (*t*_10d (11)_ = 16.432, *P* < 0.0001; *t*_20d (11)_ = 13.942, *P* < 0.0001; *t*_30d (11)_ = 27.07, *P* < 0.0001; *t*_40d (11)_ = 27.528, *P* < 0.0001), indicating that Th2-predominant immune response was induced in the immunized mice. However, it was obvious that the IgG2a was elicited after the second vaccination.

### Vaccination with rTspGST protected mice against *T. spiralis* challenge

**Fig. 7:**
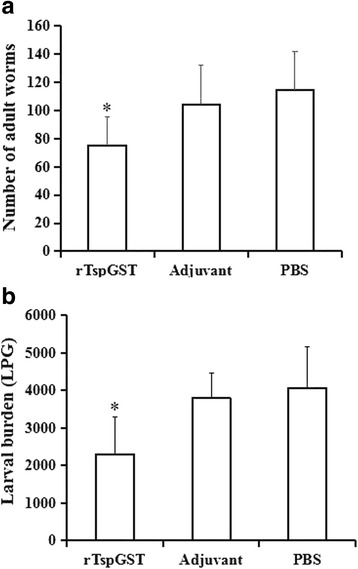
The number of adult worms in intestines **a** and larvae per gram (LPG) in skeletal muscle **b** collected from immunized mice after challenge with 300 muscle larvae of *Trichinella spiralis*. Results are expressed for each group are the mean ± SD of 10 animals each group. Asterisks indicate statistically significant differences (*P < 0.05) in adults/larvae recovered from the vaccinated group compared to adjuvant and PBS groups

Immune protection against *T. spiralis* challenge infection was evaluated in mice vaccinated with rTspGST. The mice vaccinated with rTspGST exhibited a 34.38% reduction of adult worms in intestines and a 43.70% reduction of larvae in skeletal muscles (Fig. 7) compared with the mice injected with only PBS (*F*_adults__(2,25)_ = 6.45, *P* = 0.002; *F*_larvae__(2,26)_ = 11.555, *P* < 0.0001). Moreover, the difference of adult (*t*_(16)_ = 2.524, *P* = 0.023) and larval reduction (*t*_(19)_ = 4.002, *P* = 0.001) between the vaccination group and adjuvant group was also statistically significant, whereas there was no statistical difference in the adult (*P* = 0.433) and larval worm (*P* = 0.558) burden between the alone adjuvant and PBS groups.

## Discussion

Glutathione-S-transferase (GST) is a superfamily of detoxification enzymes catalyzing detoxification reactions. The previous studies demonstrated that the GSTs of *Echinococcus granulosus*, *Fasciola hepatica*, *Necator americanus*, *Ancylostoma caninum* and *Seteria cervi* induce the evident immune protection [33–37]. The schistosome GST28 has been identified as an effective protective antigen against *Schistosoma* infections [38, 39]. The vaccination of mice with the rGST26 from *Fasciola gigantica* showed a 77 to 84% immune protection of mice against challenge infection [40]. The recombinant GSTs from *N. americanus* had powerful capacities of binding to and detoxifying the toxic heme, which contributes to the obvious immune protection against challenge infection in hamster vaccine trials [37]. The Na-GST-1 from *Necator americanus* has been developed as an important hookworm vaccine, the phase 1 clinical trials were carried out in Brazilian and American volunteers, and the prominent antigen-specific IgG responses and protective immunity were produced [41, 42]. The GST seems to be an essential protein for development and survival of the parasite and could be used as a potential target for the vaccine capable of preventing parasite infection. Our previous studies indicated that the vaccination of mice with a 24 kDa rTsGST produced a 35.71% reduction of *T. spiralis* adults in intestines and a 38.55% reduction of larvae in muscles, the vaccination with rTsGST elicited a low immune protection against challenge infection [13].

In this study, a *T. spiralis* putative GST gene encoding a 30.8 kDa protein was expressed in *E. coli* (BL21). The results revealed that the cDNA of *TspGST* was 840 bp and encoded a protein of 279 amino acids, and this protein had a molecular size of 30.8 kDa and a pI of 5.21. TspGST has only the 37% identity with TsGST at the amino acid level, suggesting that the TspGST is a significant different protein from the TsGST. The results of ELISA indicated that the immunization with the rTspGST generated the significant specific antibodies against the rTspGST, and induced the higher levels of IgG1 (Th2-predominant humoral immune responses). Anti-rTspGST antibodies recognized the native TspGST distinctly in crude proteins of AW at 3 and 6 dpi, NBL, ML and IIL, but not in the ML ES proteins. Moreover, the rTspGST could be probed by sera from mice immunized with ML crude antigens, but not by sera from mice immunized with the ML ES antigens. Nonetheless, the native TspGST in the ML crude antigens was not probed with mouse infection sera, and the ML ES antigens were not probed with anti-rTspGST sera possible because the TspGST is not an ES protein and might be a cytoplasmic protein. The results suggested that the native TsGST could not be secreted and could not elicit the host to produce an obvious humoral immune response in the process of natural *Trichinella* infection. Previous other studies also demonstrated the native TsGST was found in soluble somatic proteins of *T. spiralis* ML by Western blotting and ELISA, but not in the ES proteins of the ML [13, 43]. The results showed that the TsGST could be one component of the somatic proteins of *T. spiralis* different stages (AW, NBL, ML and IIL), but it was not from the ES protein.

The expression level of the *TspGST* gene was determined by RT-PCR and IIFT. As shown in Fig. 3, TspGST mRNA is transcribed at *T. spiralis* various stages (AW, NBL, ML and IIL). The results of IIFT with anti-rTspGST sera demonstrated that the green fluorescent staining was found in all the developmental phases (principally in cuticles, and embryos within the female uterus). The results showed that *TspGST* was expressed at all the *T. spiralis* life-cycle stages. It is suggested that TspGST mighty be an obligatory protein for the invasion, growth, and survival in a host of this nematode.

In this experiment, the Th2-predominant immune responses were elicited by vaccination with rTspGST. After being challenged orally with *T. spiralis* ML, the vaccination with rTspGST resulted in a 34.38% reduction of AW and a 43.70% reduction of ML. The results suggested that the partial immune protection produced by vaccination with rTspGST might be the results of a generation of anti-TspGST antibodies which neutralized the partial enzyme activity of GST [44, 45]. The neutralization of the GST by anti-GST antibodies could decrease the survival of the filarial nematode *Wuchereria bancrofti* in the host [46]. The GST of *Schistosoma* spp. is an ES protein, the anti-GST antibodies could sufficiently neutralize the enzyme activity of schistosome GST, which leads to prominent reduction of worm burdens in the vaccinated animals [44, 47]. But the TspGST is not a secretory protein, anti-rTspGST antibodies generated by immunization with rTspGST were not enough to neutralize TsGST enzyme activity in cytoplasm. The vaccination with rTspGST produced a partial immune protection against *Trichinella* infection. Hence, the oral polyvalent vaccines against the ES proteins of *T. spiralis* various developmental stages should be exploited in future experiments [8, 32, 48].

## Conclusion

The TsGST was expressed in all life-cycle stages of *T. spiralis* and distributed mainly in the cuticle of the parasitic nematode. The immunization with rTspGST elicited Th2-predominant immune responses and exhibited a partial immune protection against *T. spiralis* challenge infection. The TsGST could be regarded as a potential candidate target for anti-*Trichinella* vaccine, but its immune protection ought to be furtherly evaluated in a swine model, and the oral polyvalent vaccines against the ES proteins of *T. spiralis* various developmental stages should be exploited in future studies.

## Abbreviations

AW: Adult worms
DAB: 3, 3′-diaminobenzidine tetrahydrochloride
dpi: Days post-infection
ES: Excretory-secretory
GAPDH: Glyceraldehyde-3-phosphate dehydrogenase
GST: Glutathione-S-transferase
hpi: Hours post-infection
IIFT: Indirect immunofluorescent test
IIL: Intestinal infective larvae
LPG: Larvae per gram
ML: Muscle larvae
NBL: Newborn larvae
OFR: Open reading frame
RT-PCR: Reverse transcription-PCR
SD: Standard deviation
SPF: Specific pathogen-free
TspGST: *T. spiralis* putative glutathione-S-transferase
